# The identification of human pituitary adenoma-initiating cells

**DOI:** 10.1186/s40478-016-0394-4

**Published:** 2016-11-28

**Authors:** Branavan Manoranjan, Sujeivan Mahendram, Saleh A. Almenawer, Chitra Venugopal, Nicole McFarlane, Robin Hallett, Thusyanth Vijayakumar, Almunder Algird, Naresh K. Murty, Doron D. Sommer, John P. Provias, Kesava Reddy, Sheila K. Singh

**Affiliations:** 1 Michael G. DeGroote School of Medicine, McMaster University, Hamilton, ON L8S 4K1 Canada; 2 McMaster Stem Cell and Cancer Research Institute, McMaster University, Hamilton, ON L8S 4K1 Canada; 3 Departments of Biochemistry and Biomedical Sciences, Faculty of Health Sciences, McMaster University, 1200 Main Street West, Hamilton, ON L8N 3Z5 Canada; 4 Departments of Surgery, Faculty of Health Sciences, McMaster University, 1200 Main Street West, Hamilton, ON L8N 3Z5 Canada; 5 Departments of Pathology & Molecular Medicine, Faculty of Health Sciences, McMaster University, 1200 Main Street West, Hamilton, ON L8N 3Z5 Canada; 6 Pediatric Neurosurgery, Department of Surgery, McMaster Children’s Hospital, Scientist, McMaster Stem Cell & Cancer Research Institute, MDCL 5027, 1280 Main Street West, Hamilton, ON L8S 4K1 Canada

**Keywords:** Pituitary adenoma, Pituitary adenoma stem cell, CD15, Recurrence, Brain tumor-initiating cell

## Abstract

Classified as benign central nervous system (CNS) tumors, pituitary adenomas account for 10% of diagnosed intracranial neoplasms. Although surgery is often curative, patients with invasive macroadenomas continue to experience significant morbidity and are prone to tumor recurrence. Given the identification of human brain tumor-initiating cells (TICs) that initiate and maintain tumor growth while promoting disease progression and relapse in multiple CNS tumors, we investigated whether TICs also drive the growth of human pituitary adenomas. Using a nanoString-based 80-gene custom codeset specific for developmental pathways, we identified a differential stem cell gene expression profile within human pituitary adenomas. Prospective functional characterization of stem cell properties in patient-derived adenomas representing all hormonal subtypes yielded a subtype-dependent self-renewal profile, which was enriched within the CD15+ cell fraction. The tumor-initiating capacity of CD15^high^ adenoma cells was assayed in comparison to CD15^low^ adenomas using in vivo limiting dilutions, which maintained the rare frequency of TICs. Repeated analyses using sorted cell populations for CD15+ TICs compared to CD15- adenoma cells provided further evidence of xenograft tumor formation to support CD15+ cells as putative pituitary adenoma-initiating cells (PAICs). The clinical utility of our findings was established through *in silico* analyses and comparative gene expression profiling of primary and recurrent pituitary adenomas. CD15 was enriched in recurrent adenomas, which was validated using routine clinical immunohistochemistry in a limited number of samples. Our work reports the first prospective identification of human PAICs using CD15. Patients with CD15^high^ adenomas may therefore benefit from more aggressive surgical interventions and chemo/radiotherapy.

**Electronic supplementary material:**

The online version of this article (doi:10.1186/s40478-016-0394-4) contains supplementary material, which is available to authorized users.

## Introduction

Inter- and intratumoral heterogeneity are hallmark features of human central nervous system (CNS) tumors, as distinct molecular subgroups and cell populations have been described in tumors that otherwise appear histologically homogeneous [9, 29, 31]. Pituitary adenomas (PAs) account for 10% of diagnosed intracranial neoplasms and have been described in up to 25% of adult autopsies [2]. While surgery is often curative, patients with invasive macroadenomas (>10 mm) continue to experience significant morbidity due to the overexpression of pituitary hormones and compression of surrounding brain structures [2]. The anterior pituitary gland is comprised of multiple cell types that secrete unique hormones including, prolactin (PRL), growth (GH), adenocorticotropic (ACTH), luteinizing (LH), follicle-stimulating (FSH), and thyroid-stimulating (TSH) hormone [2]. These diverse cell types and their corresponding hormone expression profiles have traditionally been used to subgroup PAs for targeted therapies [2]. A subset of tumors that are non-functional or do not secrete hormones are termed null cell (NC) adenomas [2]. While the multicellular milieu and hormonal subtypes of these tumors contribute to intra- and intertumoral heterogeneity, respectively, the initiation and maintenance of human PAs remains poorly understood [3, 19, 35, 36].

Contemporary frameworks of cancer recognize a rare population of cells, termed tumor-initiating cells (TICs) that are able to initiate and maintain tumor growth [13]. By applying culture conditions and assays used to characterize normal neural stem cells (NSCs), we were among the first researchers to identify such cells from a variety of human brain tumors termed brain tumor-initiating cells (BTICs) [25, 26]. While TICs have traditionally been isolated and characterized using flow cytometric cell-sorting, no study has prospectively isolated and characterized TICs in human PAs [1, 3, 5–7, 10, 19, 35]. A unique property by which TICs may induce oncogenesis is self-renewal [23], defined as the ability of the parental cell to generate an identical daughter cell and a second daughter cell of the same or different phenotype and genotype [24]. Enhanced self-renewal as a mechanism of tumor initiation in PAs has been demonstrated with the growth of plurihormonal adenomas following the targeted expression of oncogenes in murine pituitary stem cells [1, 5, 7, 10]. The spontaneous PAs that develop in Rb^+/−^ mice provide further evidence for a hierarchical distribution of cells in these tumors [5]. Cells prospectively isolated using the surface marker Sca1 in this model represent a rare fraction of the bulk tumor with an enriched self-renewal capacity and tumor initiation potential in xenografts. Whereas TICs have been reliably isolated and characterized in murine models of PA [1, 5, 7, 10], our insight into human PAICs has been limited to observational studies in which adenoma cells are cultured as tumor spheres in serum-free media or immunohistochemical staining for the expression of putative regulators of self-renewal [3, 19, 35, 36]. While these studies may suggest an active TIC population in human PA, they have largely been limited to a minority of hormonal subtypes and have yet to perform in vivo limiting dilution assays to assess the frequency of TICs in human PA. Consequently, a functional validation of putative markers of human PAICs may yield a robust profile of these cells within treatment-refractory adenomas.

In this study, we report the use of a nanoString-based 80-gene custom codeset specific for developmental pathways to identify differentially expressed TIC markers and self-renewal genes in primary and matched-recurrent PAs. Prospective flow cytometric analysis on one hit, CD15, in additional PAs representing all functional and non-functional subtypes reliably demonstrated CD15 as a marker of adenomas with an enriched tumor sphere formation capacity. Cell-sorting for CD15 further established CD15+ cells as putative PAICs as these cells displayed a marked sphere formation phenotype and expression of genes associated with the developing pituitary when compared to CD15- cells. The tumor-initiating capacity of PAICs from CD15^high^ adenomas along with CD15+ adenoma cells was assayed using in vivo limiting dilutions, which maintained the rare frequency of PAICs. We further establish the clinical utility of our findings by demonstrating CD15 to be enriched in residual/recurrent adenomas by *in silico* analyses, gene expression profiling, and a retrospective cohort of patient samples immunostained for CD15. Our work reports the first prospective identification of human PAICs using CD15. Patients with CD15^high^ adenomas may therefore benefit from more aggressive surgical interventions and chemo/radiotherapy.

## Materials & methods

### nCounter System (NanoString) gene expression profiling

Fourteen pituitary adenomas of varying subtypes (Table 1, PA1-14) along with 2 primary and matched-recurrent pituitary adenomas (Table 2) were used to isolate RNA from formalin-fixed paraffin-embedded (FFPE) tissue using Roche High Pure FFPE RNA micro kit. Exactly 250 ng of RNA was run for each patient sample. Analysis using nCounter Gene Expression system was conducted at McMaster University’s core facility. A custom codeset synthesized by nCounter (NanoString Technologies, Seattle, WA, USA) was designed. The recommendations outlined by NanoString Technologies were all followed regarding mRNA sample preparation, hybridization, detection and scanning, and data normalization.

**Table 1:** Pituitary adenoma stem cell patient isolates: Clinico-pathological data

Sample ID	Gender	Age	Adenoma Subtype	CD15%	CD133%	Sox2%
PA1	M	69	GH	15.92	1.82	3.55
PA2	M	72	ACTH	10.25	89.75	12.82
PA3	M	35	GH	75.15	1.2	9.53
PA4	F	66	FSH/LH	83.46	16.55	4.54
PA5	M	76	GH	7.12	0.54	3.11
PA6	M	53	FSH/PRL	4.11	23.13	11.35
PA7	F	26	GH/PRL	2.59	47.98	28.36
PA8	F	52	Null Cell	62.71	1.07	2.24
PA9	F	76	LH	4.51	13.11	9.23
PA10	F	60	Null Cell	80.72	19.29	2.74
PA11	M	80	Null Cell	84.22	1.99	2.05
PA12	M	83	Null Cell	14.98	11.32	4.52
PA13	M	80	Null Cell	72.70	1.52	2.74
PA14	M	48	GH/PRL	7.06	3.92	6.63
PA15	M	51	GH/PRL	3.11	1.42	3.13
PA16	M	70	Null Cell	53.12	0.45	1.19
PA17	M	42	GH	99.56	0.47	19.24
PA18	M	64	Null Cell	7.23	12.21	7.31
PA19	M	52	Null Cell	98.86	0.96	15.07
PA20	M	72	GH	9.86	2.26	1.78
PA21	F	71	Null Cell	62.12	1.11	1.42
PA22	F	71	Null Cell	44.15	1.78	2.51

*PA* pituitary adenoma, *M* Male, *F* Female, *GH* Growth Hormone, *ACTH* Adrenocorticotropic Hormone, *PRL* Prolactin, *FSH* Follicle-Stimulating Hormone, *LH* Luteinizing Hormone

**Table 2:** Clinico-pathological demographics of matched primary and recurrent pituitary adenomas

Sample ID	Gender	Age	Adenoma Subtype	Time to Recurrence
PA23 (Primary 1)	F	49	Null Cell	7 years
PA24 (Recurrent 1)		56	TSH, FSH/LH (focal)	
PA25 (Primary 2)	M	32	GH/PRL	3 years
PA26 (Recurrent 2)		35	GH/PRL	

*PA* pituitary adenoma, *M* Male, *F* Female, *GH* Growth Hormone, *PRL* Prolactin, *FSH* Follicle-Stimulating Hormone, *LH* Luteinizing Hormone, *TSH* Thyroid-Stimulating Hormone

### Dissociation of primary human pituitary adenoma tissue and tumor sphere culture

Human pituitary adenoma samples (Table 1) were obtained from consenting patients, as approved by the Hamilton Health Sciences/McMaster Health Sciences Research Ethics Board. Briefly, samples were dissociated in artificial cerebrospinal fluid containing 0.2 Wunisch unit/mL Liberase Blendzyme 3 (Roche filtered through 70 μm cell strainer. Tumor cells were resuspended in tumor sphere medium consisting of a chemically defined serum-free tumor sphere medium (TSM), and plated in an ultra-low attachment plate (Corning). The components of our complete TSM per 500 mL include: Dulbecco’s modified Eagle’s medium/F12 (450 mL; Invitrogen), N2-supplement (5 mL; Invitrogen), HEPES (5 mL; Wisent), glucose (3 g; Invitrogen), *N*-acetylcysteine (60 μg/mL; Sigma), neural survival factor-1 (10 mL; Lonza), epidermal growth factor (20 ng/mL; Sigma), basic fibroblast growth factor (20 ng/mL; Invitrogen), leukemic inhibitory factor (10 ng/mL; Cehmicon). All PAIC patient isolates used for experimentation were not renewable cell lines, but rather minimally cultured cell isolates (24 h to <1 week) within tumor sphere conditions to select for TICs.

### Secondary sphere formation assay

After primary tumor sphere formation was noted, spheres were dissociated to single cells and replated in TSM as previously described [26]. Secondary sphere formation was calculated from the number of spheres formed from 2000 dissociated single cells.

### Quantitative real-time–polymerase chain reaction

Total RNA from samples was isolated using the Norgen RNA extraction kit (Biotek) and reverse transcribed using qScript cDNA Super Mix (Quanta Biosciences) and a C1000 Thermo Cycler (Bio-Rad). qRT-PCR was performed using the CFX96 (Bio-Rad) with SsoAdvanced SYBR Green (Bio-Rad) using gene specific primers. Data were presented as the ratio of the gene of interest to *GAPDH* (glyceraldehyde-3-phosphate dehydrogenase) as control using 2^ΔCT^. The program Primer3 (NCBI, Primer-BLAST, http://www.ncbi.nlm.nih.gov/tools/primer-blast) was used to design primer sequences provided in Table 3.

**Table 3:** qRT-PCR primers

Gene	Forward Primer	Reverse Primer
GAPDH	TGCACCACCAACTGCTTAGC	GGCATGGACTGTGGTCATGAG
Pax7	CTAAGGATGTTGAACGGGCA	TTCTCCGTTGGAACTGATGG
Sox2	CTAAGGATGTTGAACGGGCA	TTCTCCGTTGGAACTGATGG

### Flow cytometric cell sorting

Primary pituitary adenoma tumor spheres were dissociated to single cell suspension. Stemness marker expression was assessed using flow cytometric sorting (MoFlo XDP) using PE-labeled anti-CD15 (1:10, Beckman Coulter Inc., Brea, CA, USA), APC-labeled anti-CD133 (1:10, Miltenyi Biotec GmbH, Bergisch Gladbach, Germany), and v450-labeled anti-Sox2 (1:20, BD Biosciences, San Jose, CA, USA) antibodies. The percentage expression of CD15 for each group of unsorted cells and purities for CD15+/CD15- sorted cells were determined. The appropriate isotype control served as the negative control.

### In vivo PAIC injections and H&E staining of xenograft tumors

In order to achieve a human-mouse xenograft model, immunodeficient NOD-CB17-SCID mice (*n* = 16) were used for optimal engraftment of human cells according to Research Ethics Board-approved protocols. Due to the susceptibility of these mice to infection, all procedures were performed in a designated clean room and within a BSL-2 hood. Cells to be injected were re-suspended in 10 μL of PBS. Once mice were anesthesized, they were secured into a stereotactic frame. Using a cotton swab, the head of the mouse was cleaned with surgical detergent followed by water and then a providone-iodine surgical scrub. A sagittal cut was then performed down the midline of the mouse’s head using a scalpel. The cut ran from between the eyes to the point that is equidistant from both mouse ears. The periosteum of the skull was removed by scraping with the scalpel. Next, the sagittal and coronal sutures were located and the point located 2 mm posterior to the coronal suture and 3 mm to the right of the sagittal suture was identified. A burr hole was drilled at this point by tapping the drill bit lightly against the skull. The drill was applied until a reddish area was visible or until a Hamilton syringe could penetrate through the remaining skull. The 10 μL PBS solution containing tumor cells was injected using a Hamilton syringe at a depth of 3 mm of the needle head into the burr hole at an angle of either 90° or 60° from horizontal. The injection of cells was performed in one smooth, uninterrupted motion. Mice were injected with biological replicates (*n* = 4) consisting of three dilutions (1×10^5^, 5×10^4^, 1×10^4^ single-cell suspensions). 5 additional mice were also injected with PAICs sorted for CD15 (1 CD15+ mouse at 5x10^4^ cells and 2 CD15- mice, each at 5×10^4^, 1×10^5^). The end of the syringe was then tapped three times to ensure that droplets remained in the brain and were not displaced during the removal of the Hamilton syringe. The wound was then sutured with 2–3 stitches using a simple interrupted technique. An additional two extra throws after the initial knot had been tied were also used prior to cutting off any excess suture thread. 1 mL of sterile 0.9% Sodium Chloride was subcutaneously injected by inserting the syringe into the scruff of the neck or the gluteal region of the mouse. 0.5 mL of buprenorphine (Temgesic) was also injected subcutaneously using the same method and location. The mouse was then placed in a recovery cage near a heat source until it awoke. On the first day following surgery, mice were injected subcutaneously with 1 mL of sterile 0.9% Sodium Chloride and 0.5 mL of buprenorphine. Mice were then observed until experimental endpoint. All resulting human tumor xenografts were fixed, embedded in paraffin for hematoxylin and eosin (H&E) staining; images were taken using an Aperio Slide Scanner and analyzed using ImageScope v11.1.2.760 software (Aperio).

### In situ bioinformatic analysis

All data was publicly available and downloaded from the gene expression omnibus (http://www.ncbi.nlm.nih.gov/geo/). A pituitary tumor cohort (GSE26966) was used to evaluate the expression of stemness genes [16]. This cohort is composed of 23 samples comprising 8 normal pituitary samples, 9 pituitary tumors, and 5 pituitary tumor reccurences. Each sample underwent global gene expression profiling with the Affymetrix U133 Plus 2.0 microarray platform. The raw intensity files (.CEL) comprising each dataset were download and normalized using the Robust Multichip Algorithm (RMA) to generate probeset intensities [12]. When multiple probesets mapped to the same gene, the median intensity probeset value was taken to represent gene intensity. T-tests were used to compare gene expression between normal pituitaries and pituitary tumors, as well as recurrent and primary pituitary tumors.

### CD15 immunohistochemical staining of primary and recurrent pituitary adenoma

Briefly, formalin-fixed paraffin embedded patient blocks were cut at 5 μm sections and immunostained using the avidin-biotin-peroxidase complex method with diaminobenzidine as the chromogen. The primary antibodies used were mouse monoclonal antibodies against CD15 (1:100; Dako, Mississaugua, ON, Canada).

### Statistical analysis

For all in vitro studies, biological replicates from at least three tumors are compiled for each experiment in order to achieve statistical power; unique samples were not pooled before analyses. Data represent mean±s.e.m., *n* values are listed in figure legends. Student’s *t*-test analyses were performed accordingly, using the Prism 4.03 software package (GraphPad Software). The independent Student’s *t*-test was used to compare the continuous variables between two groups. The level of statistical significance was set at 0.05 for all tests.

## Results

### Human pituitary adenomas express stemness genes and contain a distinct cell population capable of sphere formation

**Fig. 1:**
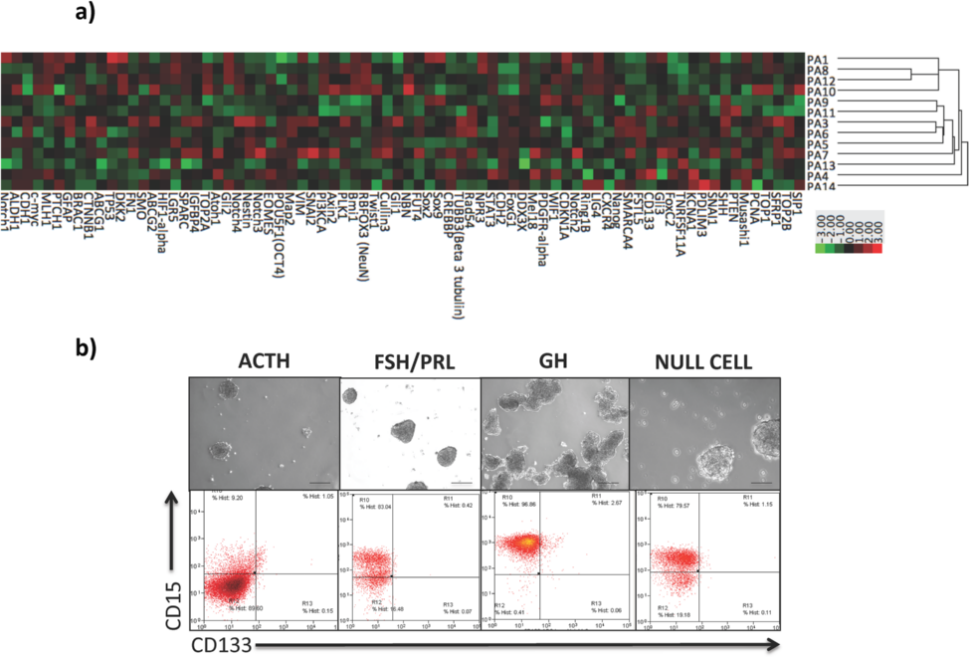
Human pituitary adenomas express self-renewal genes and contain a distinct cell population capable of sphere formation. **a** Differential stem cell gene expression profile of 14 human pituitary adenomas using a nanoString-based 80-gene custom codeset. **b** Representative photomicrograph of human PAICs and flow cytometric plots of CD15 and CD133 expression across adenoma subtypes. (×100, Scale bar = 100 μm)

Multiple reports have consistently shown genes that regulate key stem cell properties such as self-renewal and differentiation, termed stemness genes, to contribute to intra- and intertumoral heterogeneity [23, 28], while predicting clinical variables such as treatment response and overall survivorship [13]. However, no such investigations have been performed in human PAs. The successful detection of stemness genes in pituitary adenomas has previously been hampered by limitations in robust, cost-effective gene expression platforms capable of detecting genes expressed at the resolution of a single transcript, as might be the case with stemness genes, which are exclusively expressed in rare TICs [15]. Using a nanoString-based 80-gene custom codeset, we determined the differential stemness gene expression profile across 14 human PAs (Table 1, PA 1–14) representing the most common hormonal subtypes (Fig. 1a). Interestingly, our analyses revealed significant intertumoral heterogeneity across PAs, indicating a spectrum of stemness in which some PAs retained a primitive gene expression network, while others displayed a less primitive genotype.

In order to functionally validate our observed spectrum of genetic stemness, we minimally cultured primary human PAs in tumor sphere media conditions as previously described [25]. Sphere formation, a function of clonally derived TICs, was observed in all primary adenoma subtypes to varying degrees (Fig. 1b), which supported our observed genetic heterogeneity in stemness. The most compelling evidence for cancer cell heterogeneity within a given tumor is obtained by using cell surface markers to prospectively isolate distinct subpopulations of malignant cells with corresponding phenotypic diversity. However, the utility of surface markers in dissecting intratumoral heterogeneity is complicated by the reported ability of certain cancer cells to reversibly transition between phenotypic states [8]. Regardless of the presence of a rigid hierarchy, determinants of stemness have been shown to contribute to treatment failure, irrespective of whether these determinants are present within a dynamic or static population. We therefore performed flow cytometric characterization of 22 human PAs for CD15, CD133, and Sox2 (Table 1, Additional file 1: Figure S1). Although, both CD15 [22, 27, 32] and CD133 [25, 26] have previously been shown to identify TICs in highly aggressive and malignant brain tumors, their prospective flow cytometric characterization of putative PAICs has not been described. Our analyses across multiple hormonal subtypes consistently characterized a distinct CD15+ population of cells within human PAs, while CD133 marked a negligible cell fraction (Fig. 1b). Given the presence of a differential stemness gene expression profile, coupled with the capacity for sphere formation and expression of putative TIC markers, our data supports the application of the TIC model for investigating the biological heterogeneity observed in human PAs.

### Developmental genes of the normal pituitary are enriched in human pituitary adenomas, especially those adenomas with increased sphere formation

**Fig. 2:**
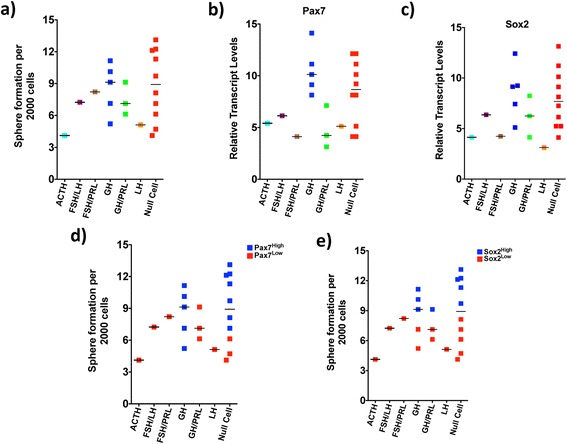
Human pituitary adenomas express genes in keeping with the developing pituitary and contain a distinct cell population capable of sphere formation. **a** Sphere formation capacity of adenomas is dependent on subtype. **b** Pax7 and (**c**) Sox2, genes activated during development of the pituitary gland are preferentially expressed in PAICs irrespective of subtype. Both, (**d**) Pax7^high^ and (**e**) Sox2^high^ tumors display an increased capacity for sphere formation when compared to Pax7^low^ and Sox2^low^ adenomas

Since several brain tumors are comprised of a functionally heterogeneous population of cells that perpetuate tumor growth through unregulated self-renewal [13, 23], we performed functional assays to assess the subtype-dependent sphere formation rate within PAs. The rate of secondary tumor sphere formation was much lower in all PA subtypes when compared to highly aggressive and malignant tumors such as glioblastoma, medulloblastoma, and brain metastasis (data not shown). However, we did observe a wide distribution in the rate of secondary tumor sphere formation across multiple subtypes, particularly, GH-secreting and null cell adenomas (Fig. 2a). While the number of adenomas within each subgroup in our analyses was reflective of their prevalence in the general population, we were limited to a single tumor for a small number of subtypes (ACTH, FSH/LH, FSH/PRL, LH), making it difficult to comprehensively interrogate the spectrum of secondary sphere formation rates among these tumors. In order to supplement our sphere formation assays, we investigated the gene expression profile of Pax7 and Sox2 within our PA cohort. Both, Pax7 [10] and Sox2 [1] have been shown to identify unique primitive cell populations in the developing murine pituitary with the potential for initiating and maintaining tumorigenesis following transformation events. Both genes contained an assorted expression profile across subtypes (Fig. 2b, c, Additional file 1: Figure S1), further highlighting the significant inter- and intrasubtype heterogeneity within PAs. Given the wide distribution in sphere formation and gene expression profile of putative PA-specific stemness genes, Pax7 and Sox2, we sought to assess the secondary sphere formation rate of Pax7^high^ and Sox2^high^ PAs compared to Pax7^low^ and Sox2^low^ PAs, respectively. Using a median cutpoint based on the gene expression values of Pax7 and Sox2, we plotted PAs according to the high or low expression of these genes relative to the rate of secondary tumor sphere formation (Fig. 2d, e). An overall trend in keeping with an increased sphere formation among Pax7^high^ and Sox2^high^ PAs was detected, which fostered the development of our framework in which a distinct population of cells within PAs across all subtypes have an enhanced ability for sphere formation, which may be regulated by the re-expression of genes present during pituitary development.

### CD15 enriches for pituitary adenoma-initiating cells

**Fig. 3:**
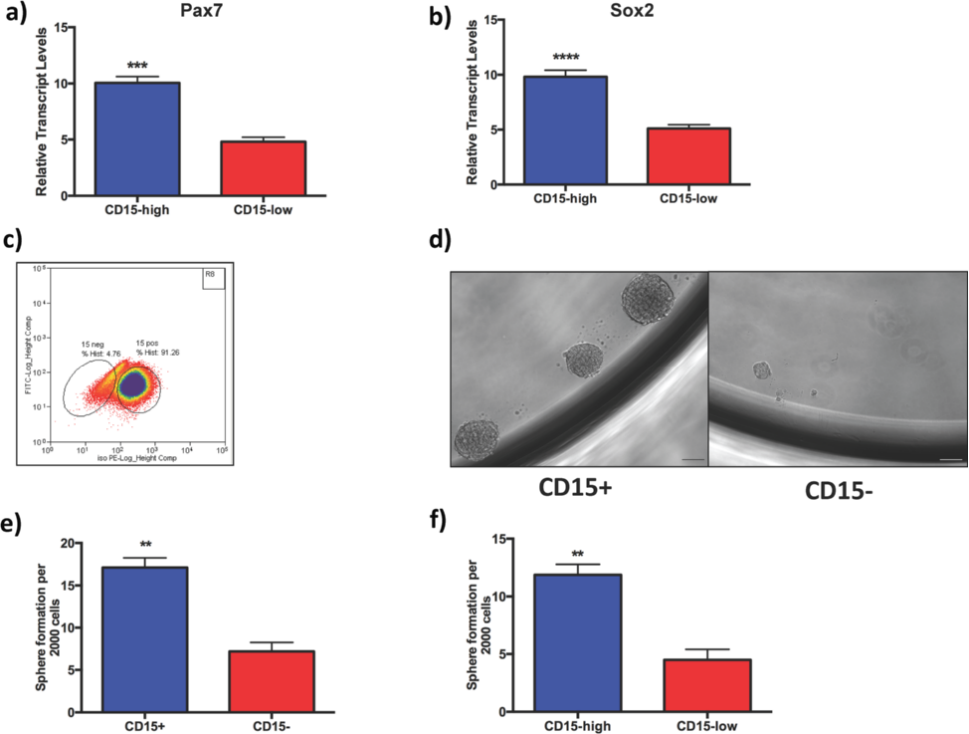
CD15 enriches for pituitary adenoma-initiating cells. Pituitary developmental genes (**a**) Pax7 (*n* = 22, *P* < 0.005) and (**b**) Sox2 (*n* = 22, *P* < 0.001) are highly expressed in CD15^high^ adenomas compared to CD15^low^ tumors. **c** Representative flow cytometric plot for enrichment of CD15+ PAICs. **d** Photomicrograph comparing sphere formation in CD15+ PAICs and CD15- cells (×100, Scale bar = 100 μm). **e** CD15+ PAICs maintain a higher sphere formation capacity when compared to CD15- cells (*n* = 2, *P* < 0.01). **f** CD15^high^ pituitary adenomas contain a higher sphere formation rate when compared to CD15^low^ tumors, irrespective of adenoma subtype (*n* = 10, *P <* 0.01)

While the ability of human PA cells to be cultured as tumor spheres and the expression of genes associated with ontology have previously been described [3, 19, 35, 36], the prospective flow cytometric characterization and isolation of putative PAICs based on cell surface marker expression has yet to be performed. Such assays provide valuable evidence in support of heterogeneous cell-cell interactions that may be critical for the evolution of tumors from ontology to oncology and throughout the course of therapy. We performed flow cytometric characterization of CD133 [25, 26], Sox2 [30], and CD15 [22, 27, 32], three markers that have reproducibly been shown to mark TICs in a number of pediatric and adult brain tumors (Fig. 1b, Table 1). Since our work consistently showed CD15 to identify a unique fraction of PA cells, we assessed the genetic and functional cellular differences between CD15^high^ and CD15^low^ adenomas. Using a median cutpoint to distinguish between CD15^high^ (*n* = 11, PA 3, 4, 8, 10, 11, 13, 16, 17, 19, 21, 22) and CD15^low^ (*n* = 11, PA 1, 2, 5, 6, 7, 9, 12, 14, 15, 18, 20) adenomas, we investigated the expression of developmental genes Pax7 and Sox2, which we found to correlate with an increased rate of secondary sphere formation (Fig. 2d, e). Both, Pax7 (Fig. 3a, *P <* 0.005) and Sox2 (Fig. 3b, *P <* 0.001) were significantly enriched in CD15^high^ adenomas when compared to CD15^low^ tumors. Therefore, we hypothesized that CD15 may mark a unique fraction of cells with an enhanced potential for self-renewal and tumor initiation. Given the technical challenges in culturing primary human PAs combined with flow cytometric cell-sorting, a limited number of PAs (*n* = 2, PA 3, 22) were sorted for CD15+ and CD15- cells (Fig. 3c). Sorted cells were plated for secondary sphere formation assays, which yielded a significant difference in the frequency of spheres in CD15+ PAICs compared to CD15- cells (Fig. 3d, e, *P* < 0.01). Similarly, CD15^high^ (*n* = 5) adenomas maintained a much higher sphere formation capacity when compared to CD15^low^ (*n* = 5) tumors (Fig. 3f, *P* < 0.01), further demonstrating the presence of a highly tumorigenic regulatory network within CD15+ PAICs. Collectively, our data illustrates the prospective isolation and characterization of a distinct cell population in human PAs, which may contribute to intratumoral heterogeneity but also distinguish tumors based on their relative expression of CD15.

### CD15^high^ adenomas and CD15+ pituitary adenoma-initiating cells are capable of tumor initiation in human-mouse xenografts

**Fig. 4:**
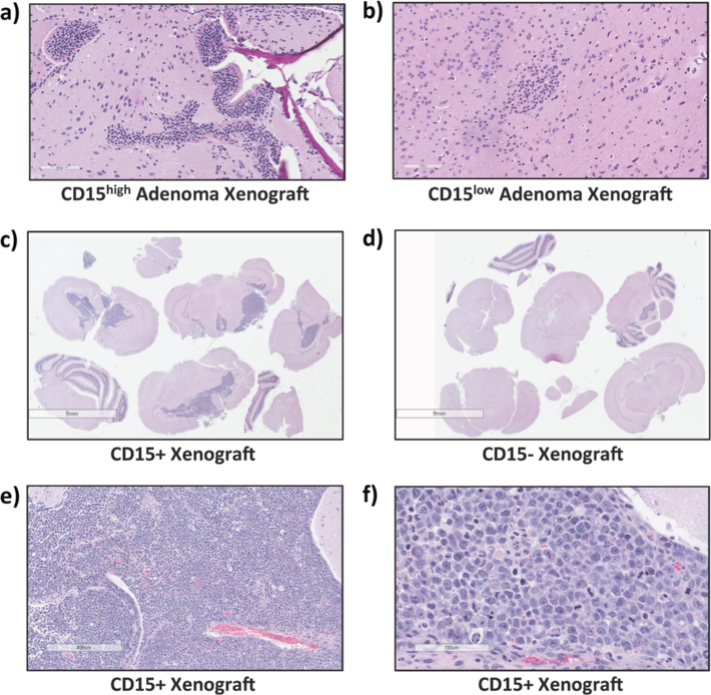
CD15+ pituitary adenoma-initiating cells maintain their tumor initiation potential in human-mouse xenografts. Representative H/E stain of pituitary adenoma xenografts generated from a limiting dilution of CD15^high^ and CD15^low^ adenomas demonstrate (**a**) tumors with a glandular morphology from CD15^high^ adenomas at a dilution of 1×10^5^ cells (*n* = 2, ×200) compared to (**b**) no apparent tumor formation in CD15^low^ xenografts at all dilutions (*n* = 2, ×200). Whole-mount H/E stain of xenografts show (**c**) CD15+ adenoma cells to result in significant tumor growth (*n* = 1, ×4), whereas (**d**) CD15- adenoma cells are unable to initiate tumor formation (*n* = 2, ×4). **e** Low (×50) and (**f**) high power (×200) images of CD15+ PAIC-generated xenografts

The most definitive method to assess the presence and function of PAICs is to carry out in vivo xenotransplantation assays and thereby quantify the frequency of tumor initiation [26]. Given the limited proliferative potential of human PA primary cultures, we initially compared the tumor-initiating capacity across the unsorted bulk population of cells isolated from CD15^high^ and CD15^low^ adenomas. Three limiting dilutions (1×10^5^, 5×10^4^, 1×10^4^) were used for intracranial injections of two biological replicates, each for CD15^high^ (PA 19, 21) and CD15^low^ adenomas (PA 18, 20). Interestingly, no tumors were formed from CD15^low^ adenomas from all three dilutions (Fig. 4b). In contrast, CD15^high^ adenomas generated small lesions at the single dilution of 1×10^5^ cells (Fig. 4a), in keeping with the rare frequency of PAICs in benign brain tumors. While this data informs us of an increased capacity for tumorigenesis within CD15^high^ adenomas, the gold standard for identifying and thoroughly characterizing TICs is through the prospective flow cytometric cell sorting of a putative TIC marker. We therefore set out to perform the first xenotransplantation assay in sorted human PA cells. Since cell-sorting for in vivo xenotransplantation assays requires a significant number of viable cells to ensure adequate engraftment, we were limited to a single biological replicate (PA 22) for this proof of principle experiment. In order to assess the frequency of PAICs in CD15+ and CD15- cells, we performed a limiting dilution consisting of 5×10^4^ (*n* = 3) and 1×10^5^ (*n* = 2) cells. We observed robust, invasive, multi-focal tumors with 5×10^4^ CD15+ cells (Fig. 4c). In contrast, no tumors were generated with 5×10^4^ and 1×10^4^ CD15- cells (Fig. 4d) and two additional mice injected with 5×10^4^ and 1×10^4^ CD15- cells were still alive at the time of writing this manuscript. Further characterization of tumors initiated by CD15+ PAICs demonstrated significant mitoses and nuclear atypia (Fig. 4e, f), in support of CD15 functioning as a highly tumorigenic cell population in human PAs. Overall, our data support the enrichment of PAICs in CD15^high^ adenomas, which is made apparent following cell sorting for CD15+ PAICs.

### Xenografts generated from CD15+ pituitary adenoma-initiating cells maintain multi-lineage specification in vivo

**Fig. 5:**
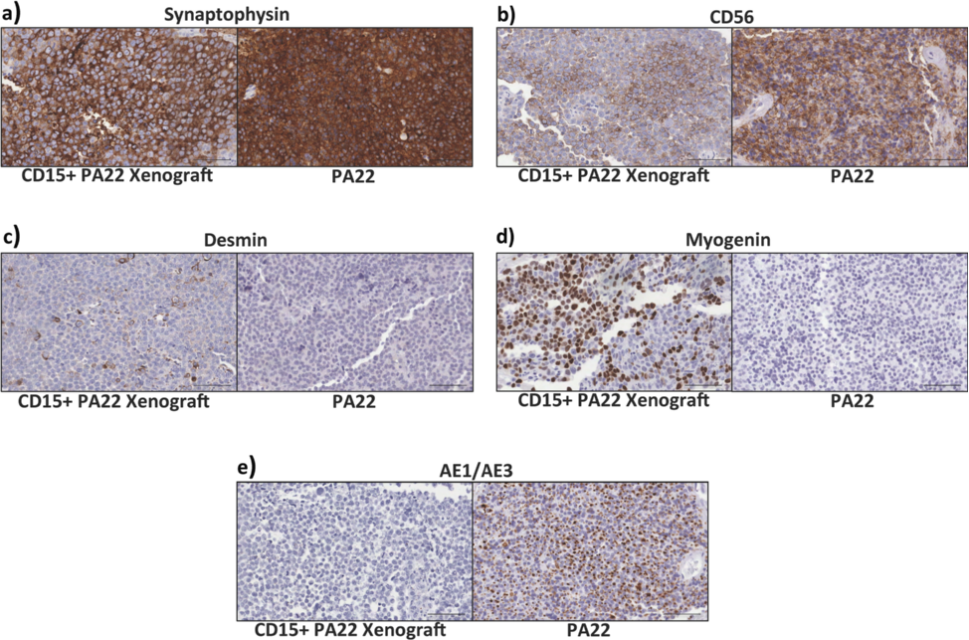
CD15+ pituitary adenoma-initiating cells promote multi-lineage specification in vivo. Representative immunostains from PA22 CD15+ xenografts (*left pane*l) and corresponding PA22 patient tissue (*right panel*). Diffuse positivity for (**a**) synaptophysin and (**b**) CD56 indicating neuroendocrine origin of tumor cells. **c** Desmin and (**d**) myogenin reactivity seen exclusively in xenografts suggesting multi-lineage specification for CD15+ PAICs. **e** Cytokeratin (AE1/AE3) reactivity present in patient tissue but absent in xenograft indicating possible clonal divergence in xenograft. All images are at magnification ×200. Scale bare = 100μm

An exhaustive immunohistochemical analysis was performed to further characterize the tumors generated from CD15+ PAICs. Given that the histological subtype of the original patient sample was a null cell adenoma (PA22), the lack of immunoreactivity within the xenograft for our pituitary hormone panel was expected (data not shown). Both samples were also immunonegative for GFAP, alpha-smooth muscle actin, EMA, and TTF1 (data not shown). In support of their neuroendocrine origin the primary patient sample and xenograft were both immunopositive for synaptophysin (Fig. 5a) and CD56 (Fig. 5b). However, desmin (Fig. 5c) and myogenin (Fig. 5d) were exclusively and unexpectedly expressed in the xenograft, highlighting the potential for multi-lineage differentiation within CD15+ PAICs. Interestingly, cytokeratin maker (AE1/AE3) expression was present in the original patient sample but was not detected in the xenograft (Fig. 5e), indicating the possibility of clonal divergence between the primary patient tumor and corresponding xenograft. Niche-specific effects may also contribute to these surprising results as our intracranial xenografts were derived from injecting cells into the frontal lobe and not the mouse pituitary. Nevertheless, previous reports of murine pituitary folliculostellate cells having the ability to differentiate into multinucleated tubular cells with immunoreactivity for myogenin [11, 17], raises the possibility of CD15+ PAICs representing a putative folliculostellate cell despite the lack of S100 expression in our xenografts.

### CD15 is highly expressed in recurrent adenomas when compared to matched primary samples

**Fig. 6:**
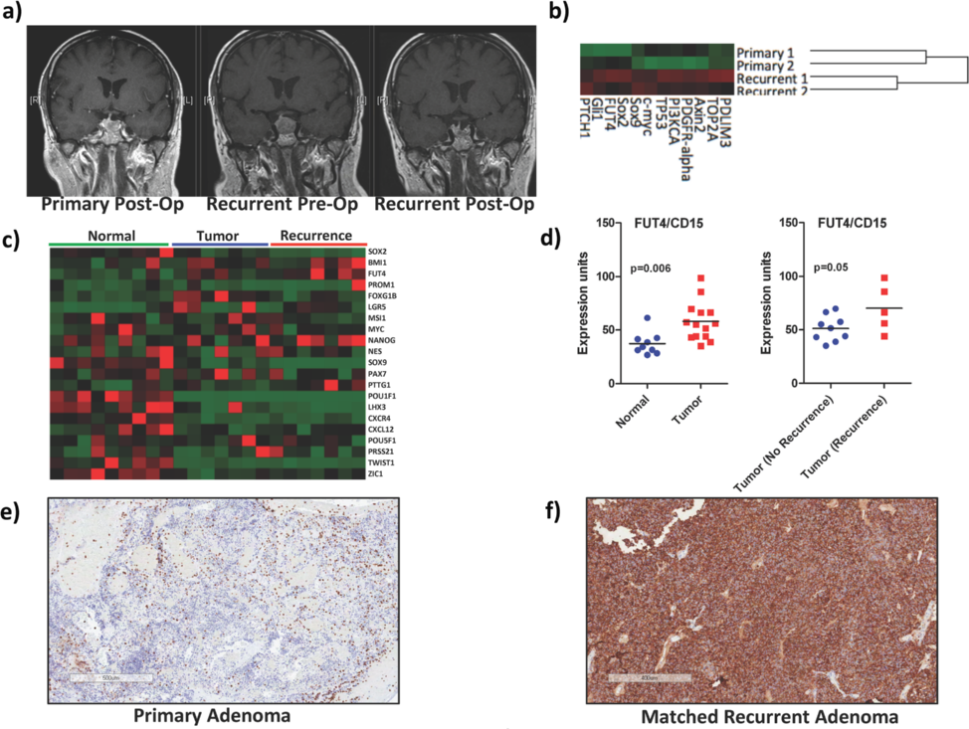
CD15 expression is significantly enriched in recurrent pituitary adenomas. **a** Representative pre- and post-operative MRI of a primary and matched-recurrent pituitary adenoma showing limited residual tumor following primary tumor resection and enhanced growth at recurrence. **b** NanoString and (**c**) *in silico* gene expression data showing enrichment of stemness genes in recurrent adenomas as compared to primary tumors. CD15 expression characterizes pituitary adenoma recurrence based on (**b**) NanoString and (**d**) *in silico* gene expression profiling. Representative CD15 stain in (**e**) primary (×50) and (**f**) matched-recurrent (×50) pituitary adenoma identifying a substantial increase in CD15 expression at tumor recurrence

While the identification of TICs has enhanced our conceptual understanding of the complex biological networks that initiate and maintain brain tumorigenesis, the clinical utility in identifying of these cells is largely dependent on their therapeutic targeting and use in prognostication as a measure of overall outcome. Since prior work has demonstrated an increasing TIC frequency to be associated with tumor aggressiveness [25, 26] and poor patient outcome [14, 20, 21], we investigated the enrichment of CD15 in recurrent/residual PAs. Given that recurrence was defined as reonset of clinical symptoms or radiological tumor growth >25%, we combined tumor recurrence and progression. We specifically assessed two primary and matched recurrent samples in which there was a significant period of time following surgery for the primary PA and the presence of a recurrent lesion (Fig. 6a, Table 2). Using our nanoString-based 80-gene custom codeset, we identified a subgroup of stemness genes that were enriched in recurrent adenomas compared to their matched primary tumors (Fig. 6b, *n* = 4, PA 23, 24, 25, 26). Of particular interest was the increased expression of components of developmental signaling pathway such as Ptch1 and Axin2, implicating the activation of these targets in driving PA recurrence. In order to validate CD15 as a clinically applicable marker of PAICs, we performed *in silico* analyses on a non-overlapping cohort of PAs for which gene expression profiles were curated for non-malignant pituitary glands, primary adenomas, and their recurrent tumors [16] (Fig. 6c). CD15 expression was able to discriminate between adenomas and normal pituitary specimens and was elevated in recurrent adenomas when compared to primary tumors (Fig. 6d).

Despite the enhanced expression of CD15 within recurrent adenomas on gene expression profiling, routine clinical neuropathology is primarily based on immunohistochemical staining. Recent work with molecular subgroups for pediatric brain tumors have attempted to distill the diagnosis of these clinically-relevant subgroups into cost-effective methods that may be readily introduced in both academic and non-academic settings [18, 34]. We therefore, performed CD15 immunostaining on a primary and matched recurrent PA as a proof of principle in describing the clinical utility of a putative PAIC marker. There was a substantial increase in CD15 expression within the recurrent tumor relative to the primary (Fig. 5e, f), illustrating the importance of combining functional in vitro and in vivo studies with novel technological platforms so that high resolution gene expression profiling may be easily translated to routine clinical immunostaining. Although, it is important to recognize CD15 enrichment in recurrent adenomas may also be due to the survival of proliferating cells or invasion of CD15+ non-resected adenoma cells. Collectively, CD15 identifies a unique population of PAICs that may drive tumor maintenance and eventual recurrence in response to multiple developmental signaling pathways.

## Discussion

Our work describes the first non-biased differential stemness gene expression profiling of human PAs complemented by functional in vitro self-renewal assays in support of a rare PAIC population. Through prospective flow cytometric cell sorting for CD15, we have identified an enhanced in vitro secondary sphere formation rate within CD15+ cells, which was reinforced by a marked increase in the turmor-initiating capacity of CD15+ PAICs compared to CD15- cells. The clinical relevance of these findings was made apparent through *in silico* analyses, gene expression profiling, and a retrospective cohort of patient samples immunostained for CD15, which collectively validated CD15 as a marker enriched in recurrent adenomas. In performing this work, we have overcome the technical limitations of culturing primary human PAs and applied our framework for investigating the biological and clinical significance of rare TICs.

Much of the literature pertaining to PAICs has been driven by work done in transgenic mouse models [1, 5, 7, 10, 33]. While several interesting observations have been made to support the derivation of PAs from targeted mutations in putative pituitary stem cell populations, the resulting lesions have also been shown to resemble human adamantinomatous craniopharyngioma [7]. In other instances, PAs derived from murine models have not fully recapitulated the hormonal heterogeneity seen in human tumors and may therefore, inadequately represent the heterogeneous cell populations that are responsible for initiating and maintaining human PAs [5, 10, 33]. The recent description of Sox2+ murine pituitary stem cells with the potential for multi-lineage differentiation and tumor propagation has offered valuable insight into non-cell-autonomous regulators of tumor growth since resulting tumors were devoid of Sox2 expression [1]. However, the clinical utility of these findings is limited since oncogenic β-catenin mutations used to propagate adenomas from Sox2+ cells have not been identified in human PAs [4]. Although transgenic murine models of PA may not effectively represent human adenomas, these models offer a homogenous critical mass of tumor cells that is essential for characterizing rare PAICs [5]. While, obtaining such a critical mass of cells required to perform repeated analyses has been a major limitation in previous human studies [3, 19, 35, 36] and in our current work, the use of non-renewable, minimally-cultured, patient-derived cells offers a model that is most in keeping with human adenomas. Technical limitations in culturing, maintaining, and expanding human patient-derived pituitary adenoma cells will need to be overcome in future studies so that the proof-of-principle CD15+ sorted in vivo experiments achieved in our work may be further characterized and validated. The effects of culture conditions in predisposing PAICs to certain cell lineages should also be considered when interpreting the multi-lineage differentiation potential observed in xenografts generated from CD15+ PAICs. We recognize the overrepresentation of non-functioning adenomas in the current study as a consequence of surgical bias pertaining to the most frequently encountered pituitary adenoma subtype in our neurosurgical department. Nevertheless, the identification of a common PAIC for all subtypes provides the greatest yield in terms of therapeutic efficacy as residual/recurrent adenomas may be collectively targeted. Our work builds on the TIC framework described in previous human studies by providing a non-biased genetic screen for stemness, postulating a developmental origin to the initiation and maintenance of PAs, and functionally validating our findings in both in vitro and in vivo assays at limiting dilutions.

## Conclusion

The failure of current cancer therapeutics may be attributed to a number of determinants such as clonal expansion based on cellular and genomic diversity, properties of stemness such as self-renewal, and the inability to effectively prognosticate those patients who require more aggressive upfront therapy. Our study provides a strategic platform for the preclinical evaluation of these factors in primary human PAs. Clinically, patients with CD15^high^ adenomas may benefit from more aggressive surgical interventions and chemo/radiotherapy as future biological studies should be focused on the identification of regulatory mechanisms that drive CD15+ PAICs to promote tumor maintenance and disease recurrence. Such efforts may reconceptualize the treatment of PA, a tumor that continues to result in significant morbidity for a substantial number of patients.

## Additional file

Additional file 1: Representative flow cytometry plots of 3 pitutiary adenoma samples showing Sox2 and CD15 expression. (TIFF 791 kb)
