# Supplementary figures and images for: The identification of human pituitary adenoma-initiating cells
Source: Acta Neuropathol Commun. 2016 Nov 28;4:125. doi: 10.1186/s40478-016-0394-4 (PMC5127041; doi:10.1186/s40478-016-0394-4)

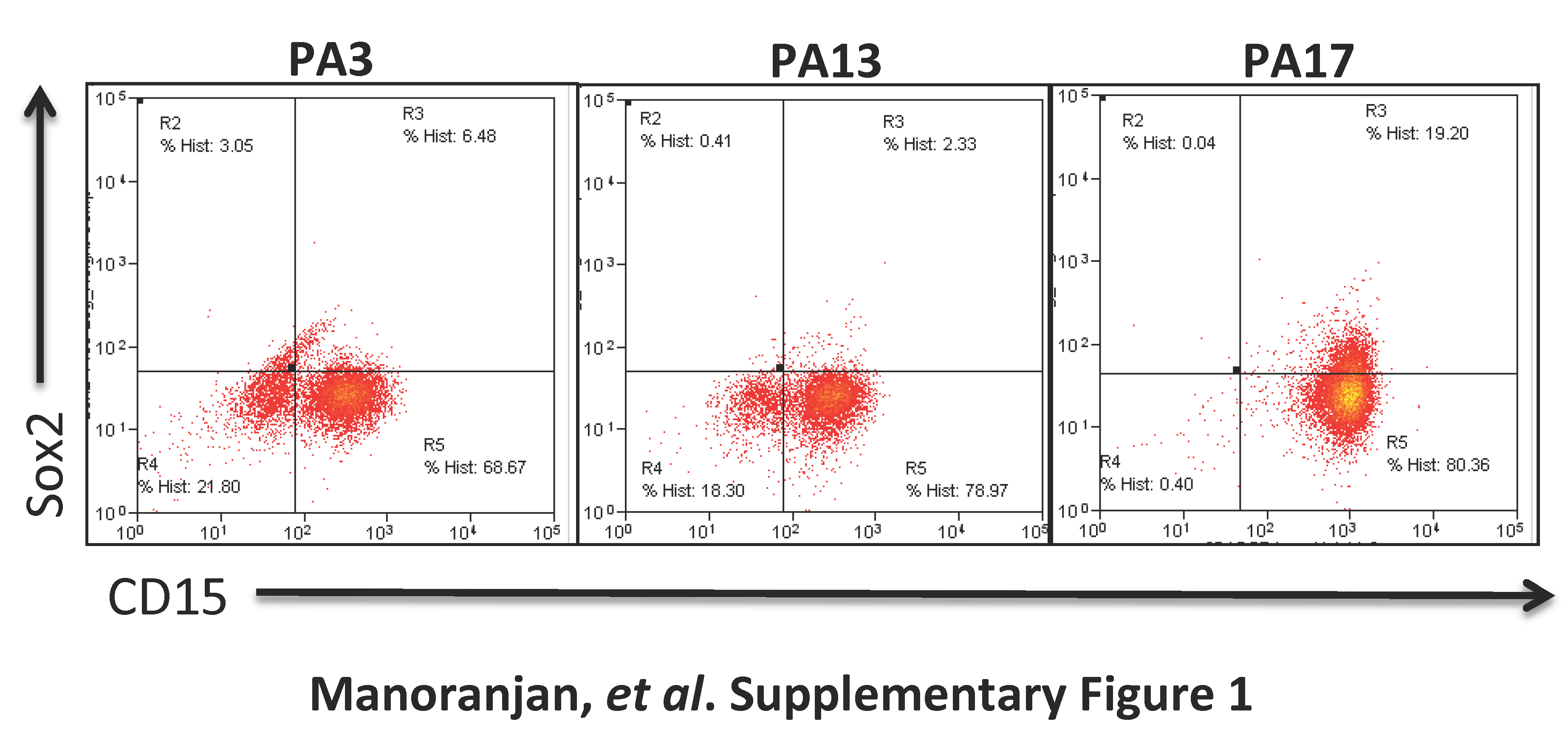

Supplement: Additional file 1: — Representative flow cytometry plots of 3 pitutiary adenoma samples showing Sox2 and CD15 expression. (TIFF 791 kb) [file 40478_2016_394_MOESM1_ESM.tiff]
